# Prognostic implications of PPL expression in ovarian cancer

**DOI:** 10.1007/s12672-022-00496-z

**Published:** 2022-05-25

**Authors:** Tian Hua, Bei-bei Zhao, Shao-bei Fan, Cai-fen Zhao, Yun-hong Kong, Rui-qing Tian, Bao-ying Zhang

**Affiliations:** Department of Gynecology, Affiliated Xingtai People Hospital of Hebei Medial University, 16 Hongxing Road, Xingtai, 054001 Hebei People’s Republic of China

**Keywords:** PPL, Ovarian cancer, Prognosis, Immune infiltrating, Bioinformatics

## Abstract

Periplakin (PPL) is a main member in plakin family, which plays important role in cellular adhesion complexes supporting and cytoskeletal integrity supplying. PPL was reported to be a potential biomarker candidate for several types of cancers. However, the biological functions and underlying mechanisms of PPL in ovarian cancer (OV) remain unclear. In the present study, we used GEPIA 2, Human Protein Atlas, Oncomine, LinkedOmics, Kaplan–Meier Plotter, STRING, CytoHubba plug-in and TIMER to determine the associations among PPL expression, prognosis, and immune cell infiltration in OV. RT-qPCR and IHC analysis were conducted to validated the role of PPL in an independent OV cohort. Compared with the normal ovary tissues, the levels of PPL mRNA and protein expression were both obviously higher in OV tumors from multiple datasets (P < 0.05), and a poor survival was observed to be strongly correlated with high PPL expression (P < 0.05). Moreover, the results were further validated by RT-qPCR and IHC analysis in an independent OV cohort. A gene-clinical nomogram was constructed, including PPL mRNA expression and clinical factors in TCGA. Functional network analysis suggested that PPL participates in the important pathways like Wnt signaling pathway, MAPK signaling pathway. Ten hub genes (LAMC2, PXN, LAMA3, LAMB3, LAMA5, ITGA3, TLN1, ACTN4, ACTN1, and ITGB4) were identified to be positively associated with PPL. Furthermore, PPL expression was negatively correlated with infiltrating levels of CD4+ T cell, macrophages, neutrophils, and dendritic cells. In conclusion, PPL may be an unfavorable prognostic biomarker candidate in OV, which was also correlated with immune infiltrating and function in immunotherapy response.

**Supplementary Information:**

The online version contains supplementary material available at 10.1007/s12672-022-00496-z.

## Introduction

Ovarian cancer (OV) carries a very poor prognosis as a progressive and aggressive gynecological malignancy [1]. It ranks fifth in cancers leading to cancer-related mortality in female patients worldwide [2]. The tumors have been spread to peritoneum as well as adjacent organs when initially diagnosed in the vast majority of patients. The 5-year overall survival (OS) is always around 25–35% for patients at advanced stage [3]. There is no denying that the late diagnosis and primary or acquired chemotherapy resistance are the main reasons for the unsatisfactory clinical outcome in OV [4]. Thus, exploring the novel effective biomarkers for early diagnosis, prediction chemotherapy efficacy and prognosis might have a much bigger positive impact on the improvement of clinical outcome in OV.

Plakin is a large family of proteins, of which many members play important roles in cellular adhesion complexes supporting and cytoskeletal integrity supplying in different tissues [5]. In cancer researches, plakin was an intriguing subject linked to many biological processes including cellular differentiation and migration, intracellular signaling, et al. [6]. In plakin family, periplakin (PPL) was a main member identified in 1997, which was organized around desmosomes in differentiated keratinocytes, and initially named 195-kD protein periplakin [7]. PPL is mainly localized in the desmosomes and inter-desmosomal plasma membranes of differentiated epidermal keratinocytes [8]. PPL interacts with other non-desmosomal proteins at the plasma membrane to regulate the signaling pathways including, Annexin 9 [9], CD64 [10] and MCHR1 [11]. In human cancer cells, PPL could also function as a localization signal in the oncogenic threonine/serine protein kinase Akt/protein kinase B (PKB)-mediated signaling [12]. The previous observations suggested that the effect of PPL expression on the function of cancer cells differ greatly, which was regulated by autocrine or paracrine factors that modulates cell cycle properties [13]. Loss of PPL may be one of the early events in the progression of oesophageal cancer [14]. PPL might act as a tumor suppressor and a new biomarker or potential therapeutic target in colon cancer [15]. While downregulation of PPL expression in vitro resulted in reduced cellular proliferation, adhesion and movement in pharyngeal squamous cell carcinomas [16]. From above all, the possible roles of PPL might be different among various cancer types. Presently, the underlying mechanisms of PPL expression in OV remain largely unknown.

In this study, our team checked the potential prognostic value of PPL in OV for the first time based on the data from the Cancer Genome Atlas (TCGA) and multiple public databases. The functional networks related to PPL in OV patients were evaluated using GEPIA 2, Human Protein Atlas, Oncomine, LinkedOmics, Kaplan–Meier Plotter, STRING, CytoHubba plug-in and TIMER analysis. RT-qPCR and IHC analysis were further conducted to validated the role of PPL in an independent OV cohort. Moreover, we developed and validated a nomogram based on the expression of PPL and clinical factors for patients with OV to better predict the prognosis of patients. Results might provide some insights in potential targets and therapeutic strategies for OV patients.

## Results

### PPL expression is up-regulated in OV

**Fig. 1:**
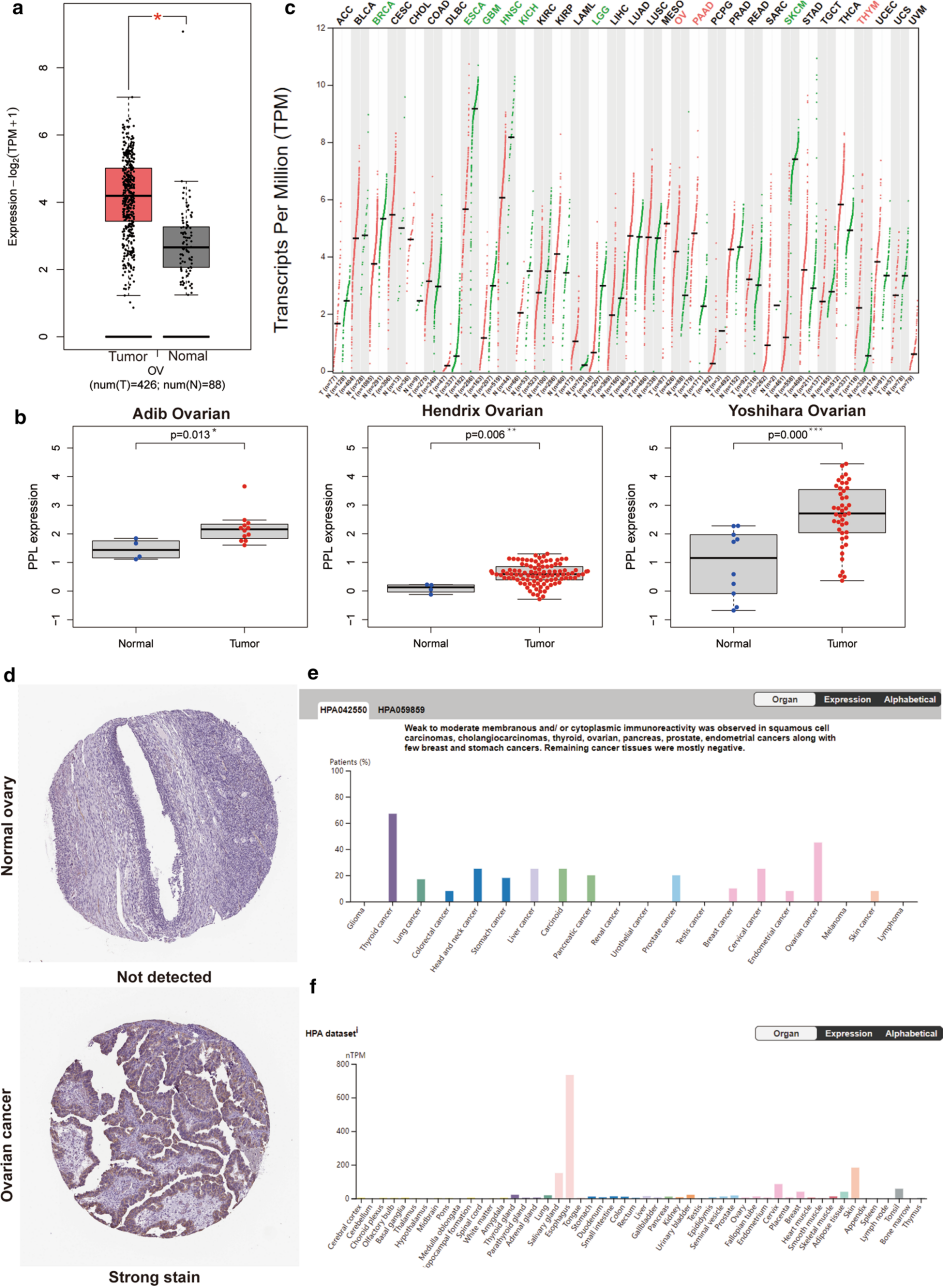
The expression PPL in ovarian cancer (OV). **a** The comparison of the transcriptional level of PPL expression between the 426 OV tissues and 88 normal tissues in TCGA cohort (*P < 0.01). **b** The PPL mRNA levels in the Adib ovarian, Hendrix ovarian, and Yoshihara ovarian datasets, respectively (*P < 0.05, **P < 0.01, and ***P < 0.001). **c** The comparison of PPL expression in pan-cancer tumor tissues and normal tissues based on TCGA and GTEx database. The cancer types with the significant higher expression comparing with the normal tissues were wrote with red colors, while the cancer types with the significant lower expression comparing with the normal tissues were wrote with green colors. The cancers wrote with black colors indicated that there were no significant differences of PPL expression between the tumors and normal tissues. **d** PPL protein was detected in OV tissues while not detected in normal tissues based on data from the human protein atlas. **e** The PPL protein expressions in multiple cancer tissues from the human protein atlas. **f** The protein levels of PPL in normal counterpart tissues

The PPL mRNA expressions from TCGA and Genotype-Tissue Expression (GTEx) projects were compared using the GEPIA 2 initially. The obvious differences were observed in Fig. 1a. (P < 0.01). The other members of plakin family, desmoplakin (DSP), envoplakin (EVPL), and bullous pemphigoid antigen 1(HLA-DRB1), the expressions of them were also significantly higher in OV tissues than normal ovary tissues (Supplementary fig. 1). Then, the Oncomine database was further used to analyze PPL mRNA expressions in OV tissues and normal ovary from several studies, which showed a higher PPL expression in OV tumors than that in normal ovary (P < 0.05, Fig. 1b).Therefore, we surmised PPL up-regulation may serve as one of the tumor drivers in OV. Moreover, we analyzed the PPL expression profile in pan-cancer, we could clearly saw that the PPL possessed higher expression trend in tumor like OV, PAAD (pancreatic), STAD (stomach adenocarcinoma), and possessed lower expression trend in tumor like ACC (adrenocortical carcinoma) and ESCA (esophageal carcinoma) (Fig. 1c), which indicated the different roles of PPL in pan-cancer. Besides the differences between OV tissue and normal ovary in transcriptome level of PPL, the examination of PPL protein level also suggested the consistent trend, showing the PPL protein level in OV tissues was higher compared with normal ovary tissues. Representative images of PPL staining in OV and normal ovary were shown in Fig. 1d download from human protein atlas data. The PPL staining was significant stronger in OV than that in normal tissues. Further, the protein level of PPL in multiple cancer tissues and normal counterpart tissues was presented in Fig. 1e and f, respectively. The higher level of PPL protein was shown in a part of cancers than that in normal tissues, such as thyroid cancer, lung cancer, and OV. Moreover, PPL could relocate protein kinase B (PKB/c-Akt)1 to different cell compartments according to the previous study. The positive correlation between PPL and AKT1 expression in OV was observed in GEPIA 2 (Supplementary fig. 2), which may provide certain evidence for the regulation role of PPL in OV.

### Elevation in PPL is associated with poor prognosis in OV patients

**Fig. 2:**
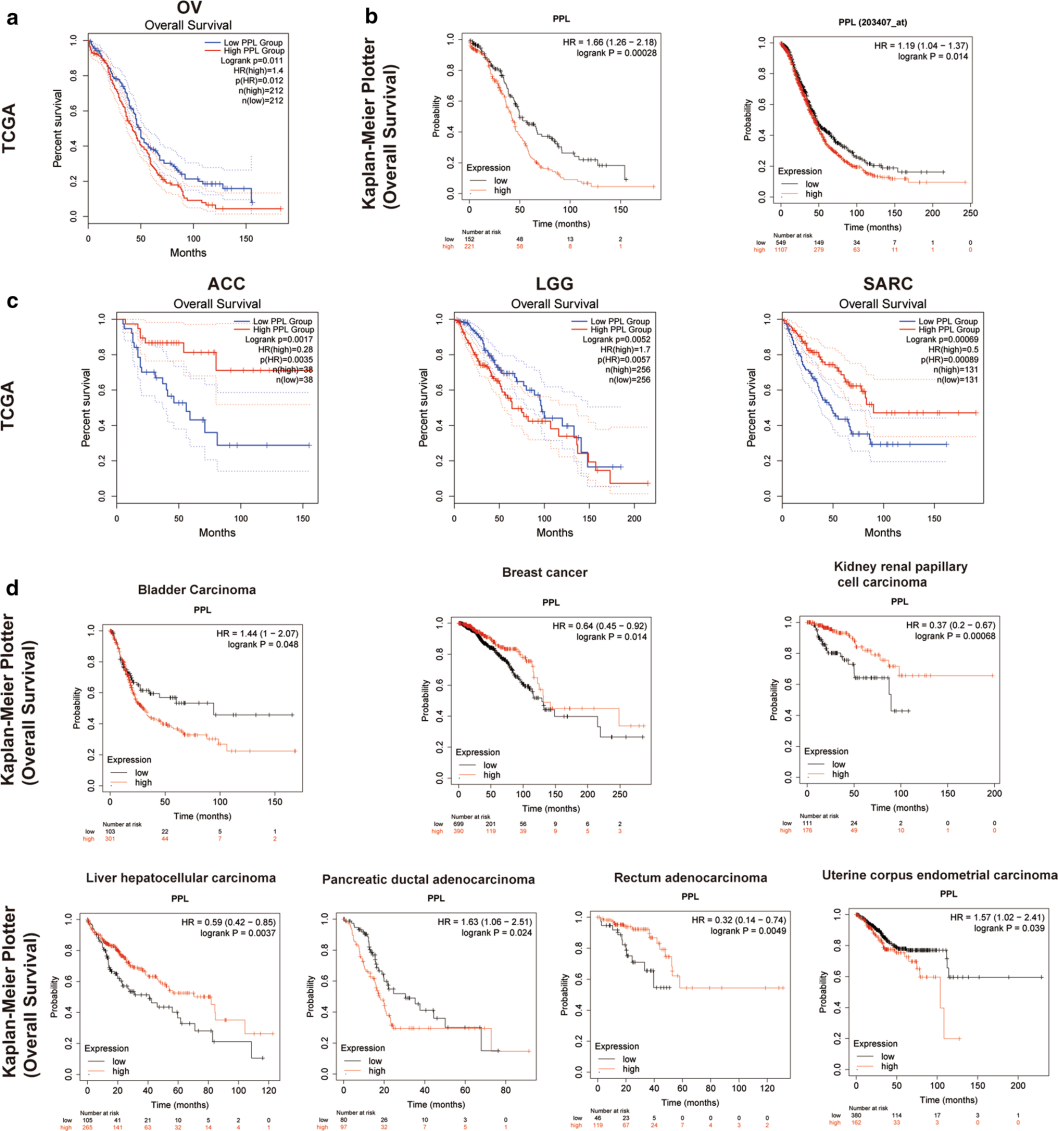
Kaplan–Meier survival curves for comparing the higher and lower expressions of PPL in multiple types cancer cohorts. **a** The survival curves in ovarian cancer (OV) from the TCGA cohort. **b** The survival curves in OV from the Kaplan–Meier plotter database analysis. **c** The survival curves in pan-cancer from the TCGA cohort. ACC (Adrenocortical carcinoma) LGG (Brain Lower Grade Glioma) SARC (Sarcoma). **d** The survival curves in pan-cancer from the Kaplan–Meier plotter database analysis

To investigate the prognostic implication of PPL in OV, Kaplan–Meier analysis was conducted based on the 326 OV patients from TCGA. The patients were grouped according to the median value of PPL mRNA expression. The clinicopathological features of 326 subjects were listed in Table 1. Patients with PPL^high^ expression presented the significant shorter OS compared with the ones with PPL^low^ expression (OS: Cox P = 0.011, HR = 1.4, Fig. 2a). Meanwhile, the consistent results in OV were shown in the survival data depending on Kaplan–Meier plotter online tool (Fig. 2b). Furthermore, the inconsistent prognosis implications of PPL expression in pan-cancer were observed. Figure 2C presented the survival curves in pan-cancer from the TCGA cohort, including ACC, LGG, and SARC. Figure 2D presented the survival curves in pan-cancer from the Kaplan–Meier plotter database analysis, including bladder carcinoma, breast cancer and liver hepatocellular carcinoma, et.al.

**Table 1:** The clinical parameters in The Cancer Genome Atlas (TCGA) ovarian cancer (OV) cohort

Characteristic	PPL expression		P-value
	PPL^high^group (n = 163)	PPL^low^group (n = 163)	
Age (year)			0.080
< 50	28	42	
≥ 50	135	121	
FIGO stage			0.931
II	9	8	
III	130	129	
IV	24	26	
Histological grade			0.295
G2	22	15	
G3	141	148	
Tumor_residual_disease			0.062
0 mm	24	41	
1–10 mm	90	79	
≥ 10 mm	49	43	

FIGO: International Federation of Gynecology and Obstetrics

### Role of PPL in an independent OV cohort

**Fig. 3:**
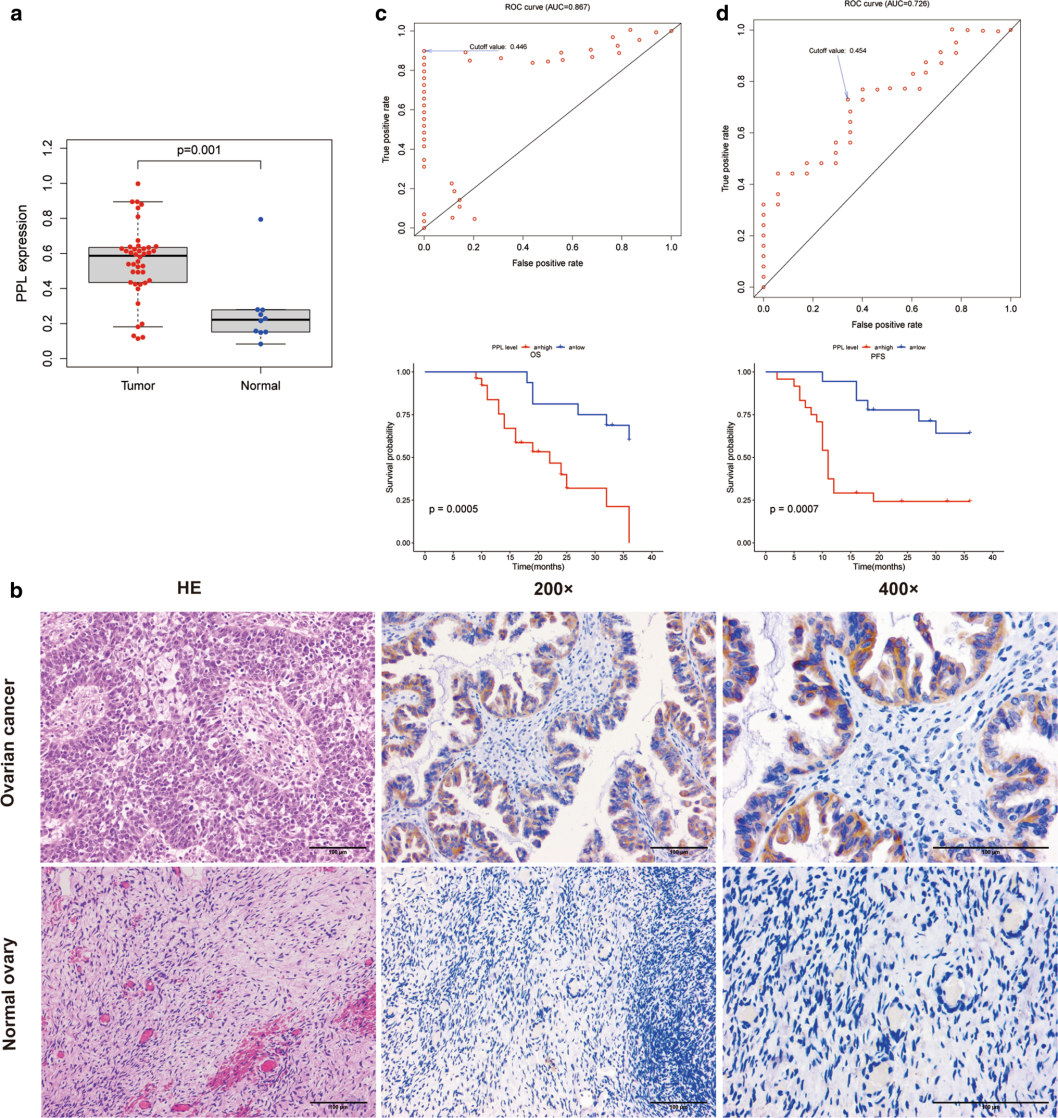
Validation of the role of PPL in an independent ovarian cancer (OV) cohort. **a** The comparison of PPL mRNA expression between OV tumor and normal ovary tissue. **b** The comparison of PPL protein level between OV tumor and normal ovary tissue. **c** ROC curve analyses based on the PPL mRNA expressions. Kaplan–Meier curves of according to Kaplan–Meier survival curve for overall survival (OS) between OV patients with the PPL^high^ and PPL^low^ expression. **d** ROC curve analyses based on the PPL expressions. Kaplan–Meier curves of according to Kaplan–Meier survival curve for progression-free survival (PFS) between OV patients with the PPL^high^ and PPL^low^ expression. P-value was calculated with the log-rank test

The clinicopathological features of 42 OV patients recruited in this study were listed in Table 2. The expression of PPL was tested in the 42 tumor samples and 10 normal ovaries, respectively. The mRNA expression and protein of PPL in OV tumors were both higher than those in normal ovary (P = 0.001, P = 0.001, Fig. 3a, b, Table 3). Representative images of PPL protein staining were shown in Fig. 3b. Among the 42 OV tumors, the positive expression rate of PPL protein was 95.23%. PPL protein was mainly expressed in cell membrane and cytoplasm. There was no significant difference of PPL protein level in tumor tissues at different FIGO stage (P = 0.930, Table 4). Further, an optimal cut-off was determined based on the receiver operating characteristic curve (ROC) analysis. As shown in Fig. 3c, we selected the point with the maximal sensitivity and specificity as the cut-off point (value = 0.436) and the patients were divided into two groups, PPL^high^ expression group and PPL^low^ expression group. The area under the ROC Curves (AUC) was 0.867. We evaluated the OS using Kaplan–Meier method and log-rank test, and the result showed that OS of the PPL^high^ expression group was significantly shorter than the PPL^low^ expression group (P = 0.0005, Fig. 3c). The area under the ROC Curves (AUC) for PFS was 0.726. The result showed the PFS of patients in PPL^high^ expression group was also significantly shorter than that in the PPL^low^ expression group (P = 0.0007, Fig. 3d).

**Table 2:** Clinical characteristics of ovarian cancer patients

Characteristics	No. patients (%)
	All patients (n = 42)
Age	
< 50	14 (33.33)
≥ 50	28 (66.67)
Pathology	
Serous	37 (88.10)
Endometrioid	5 (11.90)
FIGO stage	
I–II	18 (42.86)
III–IV	24 (57.14)
Histological grade	
G1	10 (23.81)
G2	9 (21.43)
G3	23 (54.76)

FIGO: International Federation of Gynecology and Obstetrics

**Table 3:** The differences of PPL protein levels between the ovarian cancer tissues and the normal ovary tissues

PPL expression	Ovarian cancer tissues n (%)	Normal ovary tissues n (%)	*P-*value
Negative	4 (9.52)	9 (90.00)	0.000
Weak	16 (38.10)	1 (10.00)	
Moderate	17 (40.48)	0 (00.00)	
Strong	5 (11.90)	0 (00.00)

**Table 4:** PPL protein level in ovarian cancer tissues

FIGO Stage	Positive Staining n = 38			*P*-value	Negative Staining n = 4
	Strong	Moderate	Weak		
Stage I	2	4	6	0.932	1
Stage II	1	3	4		2
Stage III	1	6	4		1
Stage IV	1	4	2		0

FIGO: International Federation of Gynecology and Obstetrics

### Evaluation and estimation of nomogram

**Fig. 4:**
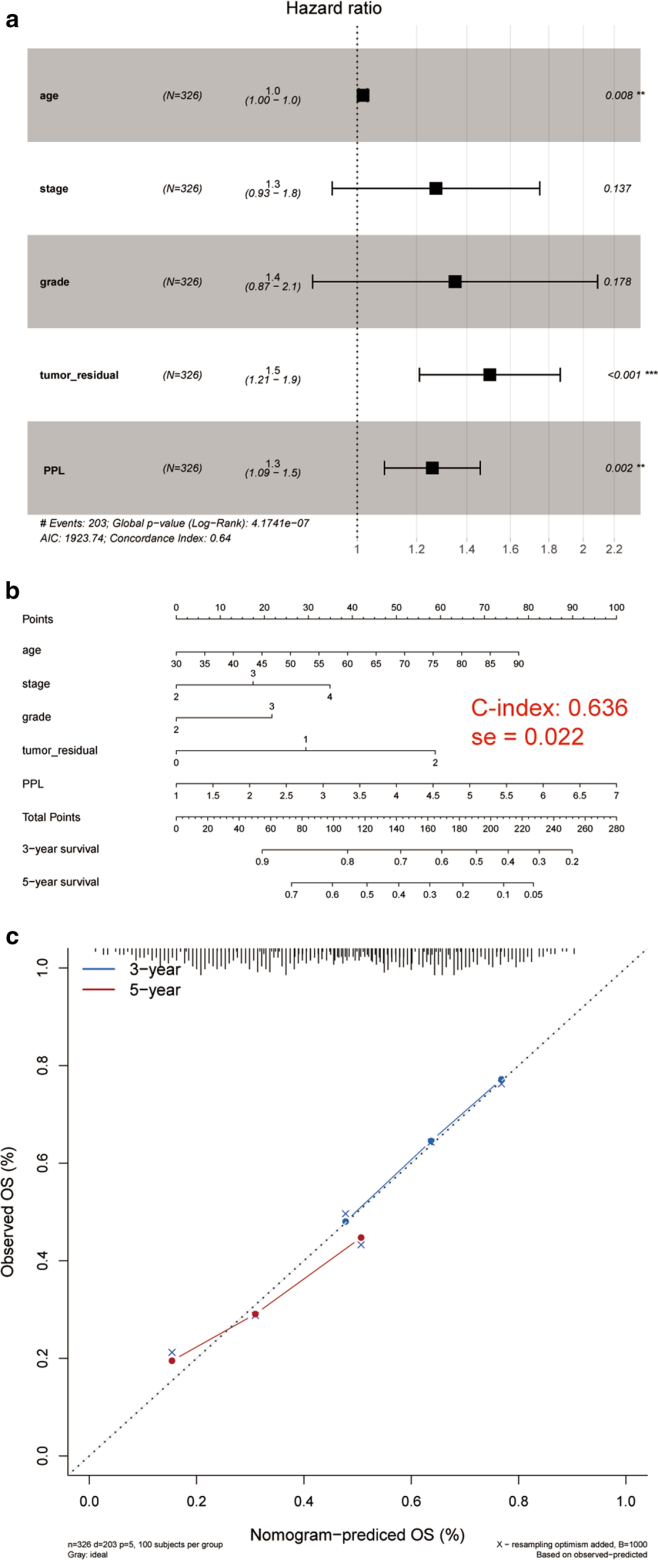
The association between PPL expression and survival outcome in ovarian cancer (OV) patients via bio-informatics analysis. **a** Forest plots for multivariate Cox analysis. se: standard error. **b** Nomogram predicting 3-year and 5-year survival. **c** Calibration curves. Calibration curves for 3-year and 5-year cancer specific survival probability depict the calibration of each model. The ideal nomogram was represented by the dashed line; The performance of current nomogram was represented by the solid line

Multivariable analysis for survival in TCGA was performed with PPL expression index included to detect whether PPL played an independent prognostic-associated role in OV patients. There was a significant association between OS and PPL mRNA expression, when tumor residual size, grade, stage and age were included in the model, (P = 0.002, HR = 1.3, 95% CI 1.09–1.5, Fig. 4a), suggesting that PPL mRNA expression may be a potential independent prognostic biomarker for OV patients. Subsequently, a gene-clinical nomogram was constructed by multivariate cox regression analysis, combined with PPL expression and clinical factors, which could visually predict patients’ prognosis according to the gene expression and clinical information, and accurately predict the survival of patients at 3 and 5 years (Fig. 4b). The point of each factor could be determined by drawing the vertical line from the variable to the point axis. By calculating the total score and locating it on the total point scale, we could get the estimated survival probability at each time point. The c-index value showed that the established nomogram had good discriminative abilities with 0.636 for survival. The calibration plots for the nomogram model indicated the predicted values by the nomogram for 3- or 5-year survival probabilities were very matched with the observed rate suggesting a strong power of this model (Fig. 4c).

### PPL co-expression networks in OV

**Fig. 5:**
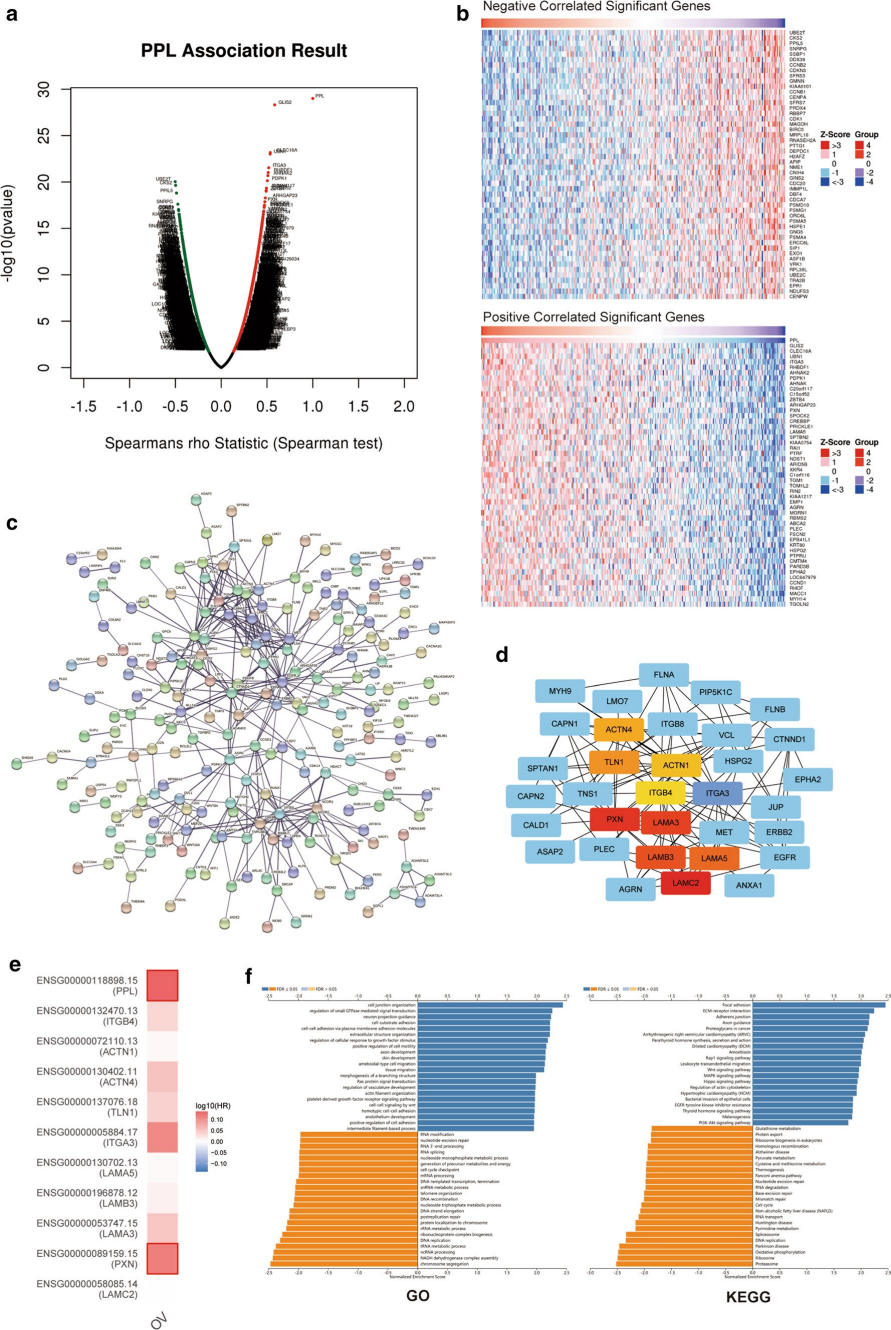
PPL co-expression genes in ovarian cancer (OV). **a** The global PPL highly correlated genes identified by Pearson test in TCGA cohort (LinkedOmics). **b** Top 50 positively and top 50 negatively correlated significant genes of PPL were presented in the heatmap. **c** A network of PPL and its co-expression genes was conducted visually using STRING in OV. **d** The hub genes (LAMC2, PXN, LAMA3, LAMB3, LAMA5, ITGA3, TLN1, ACTN4, ACTN1, and ITGB4) associated with PPL were identified in OV by CytoHubb. **e** The survival map of hub genes in OV patients (GEPIA 2). **f** Significantly enriched GO annotations and KEGG pathways of PPL in OV

To gain a deep understanding of PPL biological functions in OV, as shown in Fig. 5a, the negative and positive correlation genes with PPL were presented. Further, the top 50 positive significant, top 50 negative significant genes correlated with PPL were presented as heatmap in Fig. 5b. Figure 5c showed the STRING online database derived PPL network and its co-expression genes. The details were as follows, number of nodes: 488, number of edges: 402, average node degree: 1.62, avg. local clustering coefficient: 0.291, expected number of edges: 168, PPI enrichment P-value: < 1.0e−16. A total of 502 PPL expression correlated essential genes were identified and listed in supplementary table 1. By method MCC in CytoHubba, we found ten hub genes (LAMC2, PXN, LAMA3, LAMB3, LAMA5, ITGA3, TLN1, ACTN4, ACTN1, ITGB4, Fig. 5d). The survival map showed the hazard ratios in logarithmic scale (log10) for these ten genes and PPL in OV, the red and blue blocks denoted higher and lower risks, respectively, with an increase in gene expression (Fig. 5e). Significant GSEA annotated Gene Ontology Biological Process no Redundant term indicated that PPL co-expressed genes mainly participate in Ras protein signal transduction, cell–cell signaling by Wnt, cell cycle checkpoint, post replication repair, DNA-templated transcription, termination, etc. KEGG pathway analysis indicated the existence of enrichment in Wnt signaling pathway, MAPK signaling pathway, Hippo signaling pathway, EGFR tyrosine kinase inhibitor resistance, PI3K-Akt signaling pathway, etc. (Fig. 5f).

### PPL was correlated with immune infiltration level in OV

**Fig. 6:**
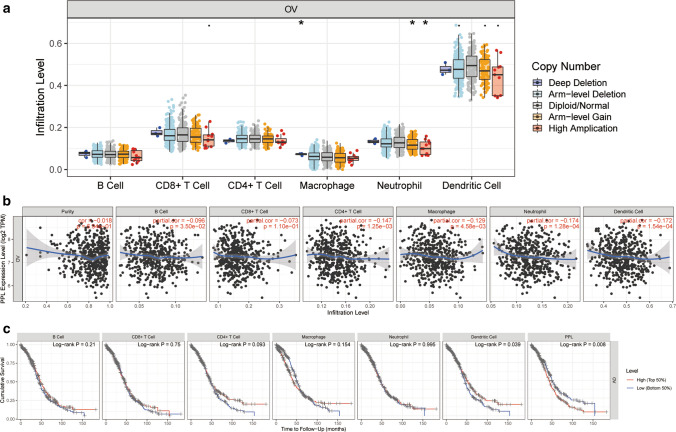
Correlations of PPL expression with immune infiltration level in ovarian cancer (OV). **a** Copy number alteration (Arm-level Deletion, Deep Deletion, High Amplication, Arm-level Gain) Correlation of PPL expression B cells, CD8+ T cells, CD4+ T cells, macrophage, neutrophil, and dendritic cells in OV. **b** Immune infiltrates in correlation with PPL in B cells, CD8+ T cells, CD4+ T cells, macrophage, neutrophil, and dendritic cells of OV (TIMER). **c** OS of B cells, CD8+ T cells, CD4+ T cells, macrophage, neutrophil, and dendritic cells processed by Kaplan–Meier analysis from TIMER. Survival differences comparisons between patients with high and low (grouped by median value) infiltrating of each kind of immune cells (*P < 0.05)

More importantly, we further assessed the underlying relationships of the mutants of PPL with immune infiltrates in OV microenvironment. A significant correlation was observed between PPL copy number alteration (CNA) and infiltrating levels of neutrophils and macrophages cells (Fig. 6a). Then, we explored whether PPL expression was correlated with infiltrating immune cells in TIMER database, the results indicated a significant correlation between PPL expression and infiltrating levels of CD4+ T cell (partial cor = − 0.147, P = 1.25e−03), macrophages (partial cor = − 0.129, P = 4.58e−03), neutrophils (partial cor = − 0.174, P = 1.28e−04), and dendritic cells (partial cor = − 0.172, P = 1.54e−04) (Fig. 6b). Furthermore, Kaplan–Meier analysis results presented a correlation between poor survival outcomes in OV and lower infiltration levels of dendritic cell (Log-rank P = 0.039), PPL gene expression (Log-rank P = 0.008, Fig. 6c). The affection of PPL expression on various immune cells including neutrophil cell, dendritic cell, CD4+ T cell, macrophage, CD8+ T cell and B cell was evaluated using multivariable hazards models based on TIMER database (Table 5). CD4+ T cell, Macrophage, Neutrophil and PPL expression were all correlated with OS based on Cox analysis. From above all, we surmised that PPL may have an impact on patients’ survival through immune infiltration interaction in OV.

**Table 5:** Multivariable hazards models evaluate the impacts of PPL expression on overall survival in the presence of infiltrating levels of multiple immune cells

cell types	coef	HR	95% CI_l	95% CI_u	*P*-value	sig
B cell	− 1.184	0.306	0.001	120.754	0.698	–
CD8+ Tcell	− 3.221	0.04	0.001	1.692	0.092	–
CD4 + Tcell	− 15.677	0	0	0	0	***
Macrophage	9.986	21,710.213	100.968	4,668,131	0	***
Neutrophil	10.407	33,105.021	5.36	204,468,000	0.019	*
Dendritic	− 1.282	0.277	0.003	26.984	0.583	–
PPL	0.359	1.431	1.187	1.726	0	***

Rsquare = 0.085 (max possible = 9.98e−01) Likelihood ratio test p = 1.84e−08 Wald test p = 2.01e−08 Score (log-rank) test p = 2.11e−08

Survival (OV) ~ variables is the formula of user-defined Cox's regression model based on TIMER database. The coefficient coef reads as a regression coefficient. HR gives you the hazard ratio, and its lower and upper 95% confidential interval are showed in 95% CI_l  and 95% CI_u

## Discussion

According to public data, the PPL expression in OV tumor was demonstrated to be significantly higher than normal ovary in this study. The higher PPL expression was, the poorer the clinical outcomes of OV patients would be, and vice versa. After that, an independent OV cohort was carried out to further validate these results. Based on PPL expression, nomogram risk score in combination with clinical characteristics was used for predicting prognosis in OV patients, which is a potential OS predicting method. What’s more, PPL expression also owned a significant correlation with immune infiltration level in OV.

As a molecular bridge for cells, plakin family members could link cell–cell junctions and intracellular cytoskeleton [6]. Besides the PPL, the other members DSP, EVPL, PLEC, and HLA-DRB1 could function in different ways to create cytoskeleton elements connection and thus forming intercellular junction complexes. For example, plectin was such a most well-studied plakin, which could interact and function in signal transduction [17]. According to the GEPIA 2 online tool, we found that the expressions of HLA-DRB1, EVPL and DSP were all significantly higher in OV tissue than that in normal ovary, with the consistent trend like PPL. The results suggested that the members of plakin family might co-express and promote carcinogenesis during OV tumorigenesis and development. Although previous research observed that the expressions of PPL were significantly lower in a variety of tumors than that in the adjacent tissues, like advanced-stage of urothelial bladder cancer and human esophageal cancers. These inconsistent results may be related to the tumor tissue specificity and heterogeneity. Moreover, an interaction between PPL and PKB/c-AKT1 has been reported in the previous research, suggesting PPL could relocate it to different cell compartments [18]. During anti-apoptosis, PKB signaling may also present an important function. Besides docking site, PPL has other roles to play, like shuttle of PKB delivery to different sub-cellular compartments after PKB was activated [12]. We could find the aberrant expression of AKT1 in many cancers including gastric, pancreatic, ovarian, lung, and breast carcinoma [19, 20]. The positive correlation between PPL and AKT1 expression might explain a possible mechanism by which PPL participates in the occurrence of OV is through the regulation in AKT1-associated signal.

PPL function network was also conducted to explore the PPL co-expression and related signaling events, thus we could further reveal the underlying mechanisms of PPL in OV pathogenesis. Totally, 502 PPL expression-correlated essential genes were identified via STRING analysis, indicating that the proteins are at least partially biologically connected, as a group. Further, the ten hub genes (LAMC2, PXN, LAMA3, LAMB3, LAMA5, ITGA3, TLN1, ACTN4, ACTN1, ITGB4) were identified to be positive associated with PPL. Of them, LAMC2, LAMA3, LAMB3, and LAMA5 are the members of laminin family. Dysregulated cell laminin interactions were major features of multiple cancers [21]. A recent study pointed out the higher mRNA expressions of LAMC2, LAMB3 and LAMA5 were observed in OV tissue compared with normal ovary. LAMC1 and LAMA5 might be prognostic factors and serve as important tumor oncogenes in OV [22]. LAMA3 was also proved to be an unfavorable prognosis biomarker in OV from another study [23]. Moreover, LAMA1/A5 and LAMC1 were significant negatively correlated with tumor immune infiltrates in OV, especially with dendritic cells, CD8+ T cells or neutrophil [22]. ITGA3 and ITGB4, belonging to integrin, might synergistically or independently regulate cell adhesion and proliferation in OV and play an important role in the prediction of prognostic for advanced OV patients [24]. TLN1 played an essential role in integrin activation, which was correlated with a metastatic phenotype of malignant tumors, such as breast cancer, hepatocellular carcinoma, and prostate cancer [25, 26]. PXN, as an important part of the focal adhesion complex, was correlated with poor clinical outcomes in patients with tumors [27]. Chen et al. [28] reported that abnormal PXN expression was related to poor prognosis, immune infiltration, and protein phosphorylation in different tumor types. Upregulated PXN expression was linked to shorter OS in OV from public data platform. In ovarian clear cell carcinogenesis, ACTN4 overexpression and genomic gain of ACTN4 may be the early molecular events [29]. This supportive information enhanced the understanding of the important role of PPL in OV. Possible molecular mechanisms of PPL involved in OV invasion and progression still need to be further explored by investigations in future.

Furthermore, for the first time, the OV patients with higher PPL expression presenting poorer prognosis were observed from multiple cohorts and the results were validated in independent 42 OV patients. Thus, determination of PPL expression in surgical specimens of OV could aid in identifying and predicting the prognosis. What’s more, the PPL prognostic landscape was visualized in pan-cancer via public databases. Higher PPL expression also shown a correlation with poorer prognosis in uterine corpus endometrial carcinoma, pancreatic ductal adenocarcinoma, as well as bladder cancer, while the low expression of PPL was correlated to poor survival in ACC, SARC, and breast cancer, kidney renal papillary cell carcinoma, et al. PPL might play different roles in different types cancer. Besides, the heterogeneity of patients provided by TCGA dataset, particularly too many varieties on the patients’ treatment plan, might contribute the inconsistence.

OV immunotherapies have been attracted increasing interest based on the results that improved clinical outcomes were associated with tumor infiltrating lymphocytes [30, 31]. Thus, for developing new immunotherapeutic strategies, a deeper insight into biological mechanisms and understanding of interaction between immunology and cancer are needed. In OV patients, PPL expression was negatively correlated with infiltrating levels of CD4+ T cell macrophages, neutrophils, and dendritic cells, which may provide power proof of the associations between PPL expression and immune markers. Also, to elucidate the important role of PPL in immunotherapy, more studies were still needed in the future.

From above all, our research studied the clinical implications of PPL expression in OV for the first time. High PPL expression was proved to be a potential unfavorable biomarker for OV. What’s more, multiple cohort studies also validated its prognostic value in pan-cancer. A potential correlation between immune cells and PPL expression implied that PPL might own the potential of crucial function in tumor immune microenvironment. However, there were several limitations in this study. First, although the study involved a bioinformatic analysis of PPL, there were no in vivo or in vitro experiments to validate the results. Therefore, future researches aiming at exploring underlying mechanism of PPL associated signaling pathways are required in further experiments. Moreover, an inadequate number of OV patients in the validate cohort enrolled in this study may be another limitation. And more precise study like prospective and multi-center study with bigger sample size should be carried out in the future to further verify these results.

## Conclusions

In this study, we explored the prognostic implications of PPL in OV patients for the first time. Our data suggested PPL might be an unfavorable independent prognostic biomarker candidate in OV. Moreover, our results suggested that PPL co-expression genes in OV might likely have far-reaching effects in pathway like Wnt signaling pathway, MAPK signaling pathway. PPL was also correlated with immune infiltrating and might play an important role in immunotherapy response.

## Materials and methods

### GEPIA2 analysis

Gene expression profiles from the GTEx projects and TCGA dataset were analyzed using the GEPIA 2 database (http://gepia2.cancer-pku.cn) [32]. Normal and cancer tissues from 33 cancer types were analyzed for PPL expression using "Dot plot" function of GEPIA2. Log2(TPM + 1) scale was used to present gene expression.

### Oncomine analysis

Oncomine (www.oncomine.org), is a cancer microarray database and web-based data-mining platform, contains 715 gene expression data sets and data from 86,733 cancer tissues and normal tissues. These datasets support diverse analyses methods including meta-analysis, interactome analysis as well as molecular concepts analysis [33]. Therefore, the Oncomine database was adopted to verify PPL expression in OV. Expression level analyses were done by plotted in R using data acquired from Oncomine database.

### The human protein atlas (HPA)

The HPA (https://www.proteinatlas.org/pathology) database contains proteomic and transcriptomic data from organs, tissues as well as cells. All these specimens are obtained from pathological or normal human tissues using immunohistochemistry (IHC) and RNA sequencing (RNA-Seq) analysis, herein, HPA database was adopted in this study to obtain comparative IHC data of PPL protein in normal ovary and OV patient tissues [34]. In addition, the information of PPL protein in the other types tumor and normal counterpart tissues were also recorded.

### Kaplan–Meier plotter database analysis

We analyzed the prognostic value of PPL mRNA expressions for OV patients using Kaplan–Meier plotter (http://www.kmplot.com), an online database containing gene expression data and survival information. In the present study, we evaluated the PFS and OS of patients for over 5-years with a Kaplan–Meier survival plot, including the hazard ratio (HR) with 95% confidence interval (CI) and log-rank P-value [35].

### Nomogram construction

The clinical data of total OV patients included in this study was downloaded through the University of California Santa Cruz Xena (UCSC Xena) (https://xena.ucsc.edu/platform, supplementary table 2). Inclusion criterion: OV patients with surgical resection; Complete clinical data; Available RNA-sequence data. Exclusion criteria: No surgery was performed; Incomplete clinical data; Lost to follow up; Incomplete RNA-sequence data. For each of the potential risk factors that constructed the nomogram based on pre-processing PPL expression which was obtained from UCSC Xena, the Cox proportional hazard regression was performed to calculate the hazard ratio (HR), as well as the corresponding 95% confidence interval (CI). Nomogram and calibration plots were performed using P-value, HR, and 95% CI of each variable of forest plot obtained from ‘forestplot’ R package. ‘rms’ package from R. Nomogram could predict patient prognosis through integrating different prognostic factors to produce personalized clinical event probability. The calibration curves were used for assessing nomogram-predicted 3-,5-year survival with observed 3- and 5-year survival, we also calculated the c-index of the with function coxph.

### Pathways interaction analysis building and protein interaction network (PPI)

In this study, the LinkedOmics database [36] (http://www.linkedomics.org/login.php) was employed for PPL co-expression analysis based on Pearson’s correlation coefficients. Heatmaps and volcano plots were used to plot the results. LinkInterpreter module from LinkedOmics was used to conduct analysis of GO_BP, KEGG pathways enrichment by the gene set enrichment analysis (GSEA). The rank criterion was P-value < 0.05 and 1000 simulations were performed. SRTING (v11.5, http://string-db.org/) [37] is a protein–protein interaction networks functional enrichment analysis online tool, we selected the organism as human sapiens, and input the positive correlated genes with PPL, then a cutoff of 0.4 for minimum interaction score was set to obtain biological functions, with disconnected nodes hidden from network. After that, Cytoscape3.9.0[38] was applied to visualize the interaction network of these proteins, hub genes(filtering degree ≥ 10) were also acquired using CytoHubba plug-in.

### TIMER database analysis

The Tumor Immune Estimation Resource (TIMER) (https://cistrome.shinyapps.io/timer/) could be used to evaluate tumor-infiltrating immune cells in a diversity of cancer types [39]. The TIMER includes over 10,000 samples from TCGA in diverse cancer types. The abundance of immune infiltrates was calculated using a partial deconvolution linear least square regression method. Based on "Survival" and "SCNA" module of TIMER database, the mutation types of PPL (including copy number alteration) with immune infiltrates in OV patients were further evaluated.

### Sampling of tissue specimens

Between Jan. 2018 and Jan. 2019, we collected the tissue samples from 42 OV patients when they received first surgery. Patient characteristics are listed in Table 2. The follow-up was regularly carried out for the following three years. Survival status of the OV patients was evaluated by OS and PFS. 10 normal ovarian epithelial tissues were gathered from patients received adnexectomy due to the gynecological benign diseases concurrently. The Ethics Committee of Affiliated Xingtai People Hospital of Hebei Medial University reviewed and approved our study (2018 [05]). Written informed consent was provided by all patients.

### The analysis of PPL mRNA and protein in an independent OV cohort

TRIzol reagent (Generay Biotech, China) was used to extract total RNA, based on manufacturers protocol. Using Revert-Aid First Strand cDNA Synthesis Kit (Thermo Scientific, U.S.A.), 500 ng total RNA was used to synthesize cDNA. With GAPDH as housekeeping gene and primers bought from Sangon Biotech Co. Ltd. (Shanghai, China), QuantiNova TMSYBR® Green PCR Kit (Qiagen, Hilden, Germany) was applied for reverse transcription quantitative PCR(RT-qPCR). Custom primers for PPL (forward: TGCAGACCCGGAGCATCTCT reverse: CCTTCTGCAGGGTCA.

CGTCC) was acquired from Sangon Biotech Co. Ltd. (Shanghai, China). Rabbit momoclonal [ERP8296] to Periplakin (ab131269, Abcam, Cambridge, UK,) was applied to detect PPL protein in the tissue samples. The predicted location for PPL was considered in intracellular. Specifically, the definition of strong, moderate, weak and negative staining was nuclear staining of > 75%, 25%-75%, < 25% and no nuclear staining of cells, respectively.

### Statistical analysis

R software v3.6.3 (R Foundation for Statistical Computing, Vienna, Austria) was applied for all statistical analysis. Pearson's chi-square test or Fisher's exact test was applied for analyzing qualitative variables. Quantitative variables analyses were performed using Wilcoxon rank-sum test (for unpaired samples). ROC was constructed using the survivalROC package in R and the optimal cut-off point was determined with the maximal sensitivity and specificity[40]. The Kaplan–Meier survival analysis and log-rank test. Kruskal–Wallis test was carried out for normal multiple groups. If not specified above, P < 0.05 were statistically significant.

## Supplementary Information

Supplementary file 1 (TIF 1130 KB)** Supplementary fig. 1.** The comparison of the transcriptional level of genes from Plakin family in ovarian cancer (OV) based on TCGA and GTEx database from GEPIA2. (a) DSP (b) EVPL (c)HLA-DRB1 (d)PLEC (* P < 0.01).

Supplementary file 2 (TIF 859 KB)** Supplementary fig. 2.** Correlations of PPL expression with AKT1 transcriptional level in ovarian cancer (OV) patients from GEPIA2.

Supplementary file 3 (XLSX 42 KB)** Supplementary table 1.** A total of 502 PPL expression correlated essential genes were identified from Linkedomics.

Supplementary file 4 (XLSX 382 KB)** Supplementary table 2.** The clinicopathologic data for the ovarian cancer patients from TCGA cohort.

## Data Availability

The data sets generated and/or analyzed during the current study are not publicly available, but are available from the corresponding author on reasonable request.

## Abbreviations

ACC: Adrenocortical carcinoma
CI: Confidence interval
DSP: Desmoplakin
EVPL: Envoplakin
HR: Hazard ratio
HLA-DRB1: Bullous pemphigoid antigen 1
HPA: Human Protein Atlas
IHC: Immunohistochemistry
GSEA: Gene set enrichment analysis
GTEx: Genotype-Tissue Expression
OS: Overall survival
OV: Ovarian cancer
PLEC: Plectin
PFS: Progression-free survival
PKB: Protein kinase B
PPI: Protein Interaction Network
PPL: Periplakin
RT-qPCR: Reverse transcription quantitative PCR
SARC: Sarcoma
TCGA: The Cancer Genome Atlas
TIMER: Tumor Immune Estimation Resource
