# Supplementary figures and images for: Prognostic implications of PPL expression in ovarian cancer
Source: Discov Oncol. 2022 May 25;13:35. doi: 10.1007/s12672-022-00496-z (PMC9133299; doi:10.1007/s12672-022-00496-z)

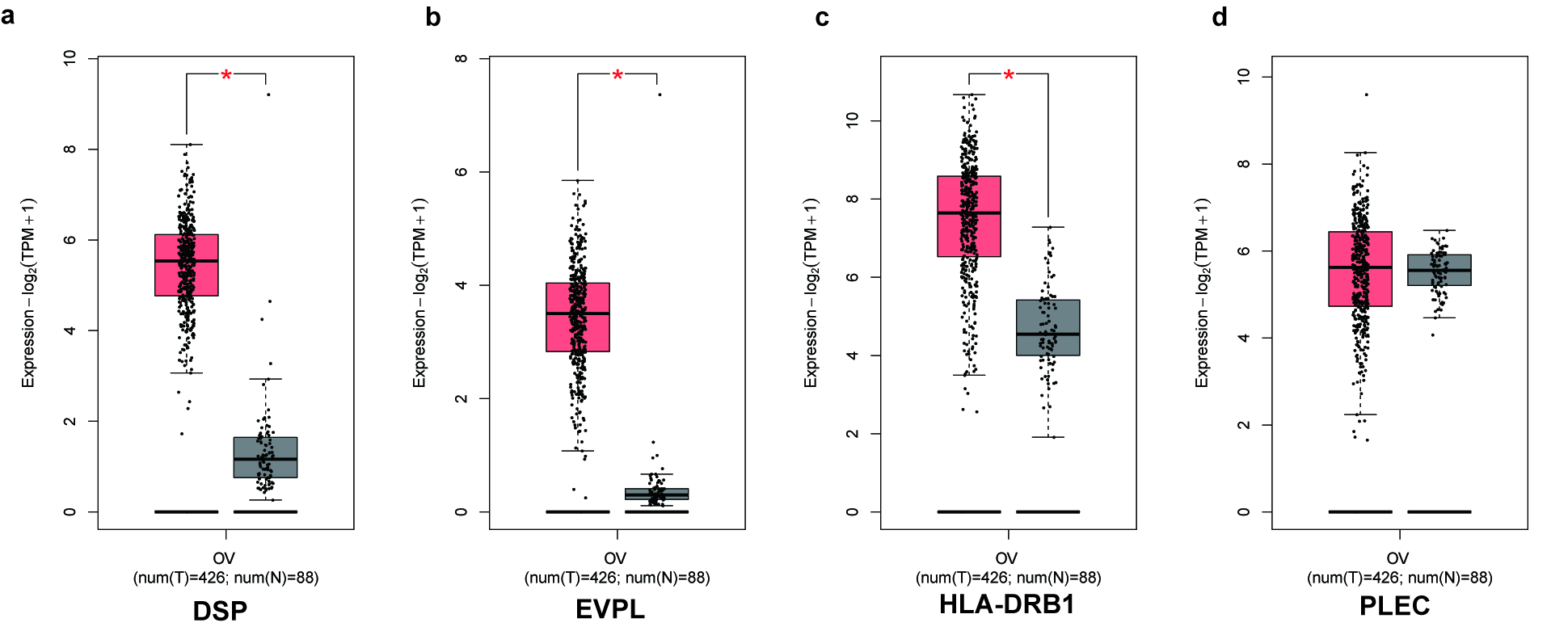

Supplement: Supplementary file 1 — (TIF 1130 KB) Supplementary fig. 1. The comparison of the transcriptional level of genes from Plakin family in ovarian cancer (OV) based on TCGA and GTEx database from GEPIA2. (a) DSP (b) EVPL (c)HLA-DRB1 (d)PLEC (* P < 0.01). [file 12672_2022_496_MOESM1_ESM.tif]

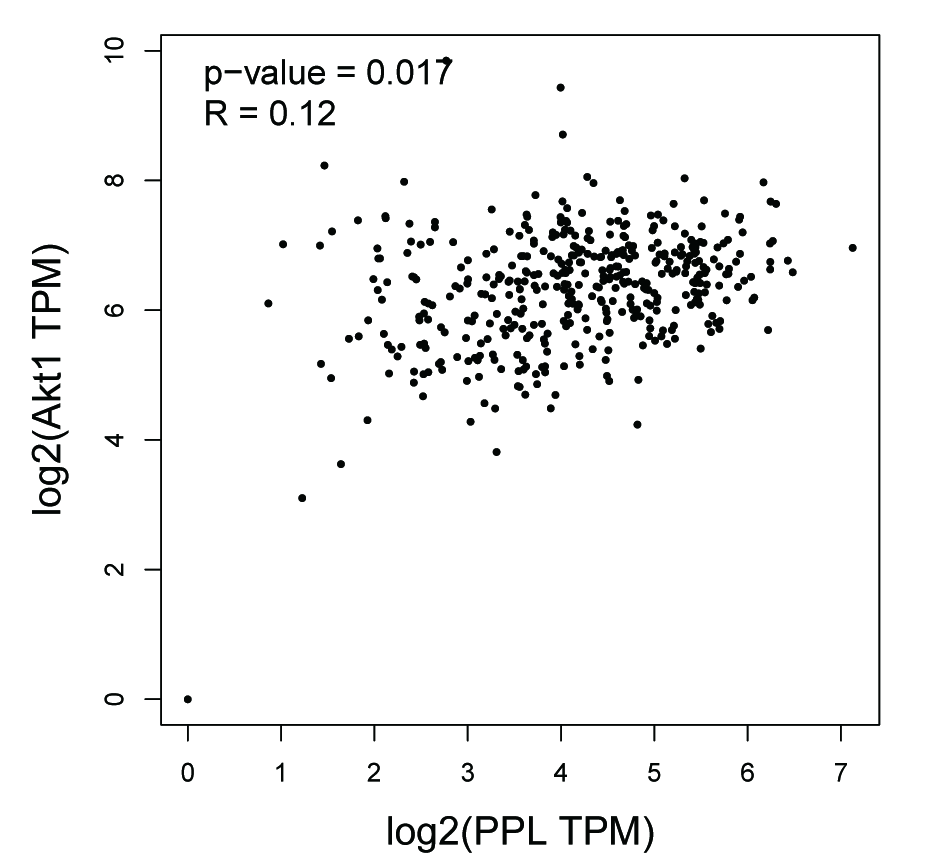

Supplement: Supplementary file 2 — (TIF 859 KB) Supplementary fig. 2. Correlations of PPL expression with AKT1 transcriptional level in ovarian cancer (OV) patients from GEPIA2. [file 12672_2022_496_MOESM2_ESM.tif]
